# Cervantes, an Honorary M.D.

**Published:** 1861-07

**Authors:** 


### Editors' Table.

Cervantes, an Honorary M.D.—"Don Quixote" is not the home-book of the Spanish people only; it has been translated into all the languages of the civilized nations of the world, and is read with avidity and delight in all of them. Of those who take it up for the first time, few know, or, if knowing, care about the intention of Cervantes in writing the adventures of the renowned knight of La Mancha and of his squire Sancho Panza. All its readers enjoy the comic, ridiculous, and extravagant incidents in the career of the two worthies; some feel the humor in addition to the fun, and a more select number appreciate the wit and the satire which run through the whole work. The design of the author was not to make knight-errantry ridiculous, but to cover with ridicule the prevailing literature of the time, in Spain, which was made up, to a great extent, of trashy romances of chivalry, in which were accumulated the grossest blunders in history, in geography, and in philosophy, with the addition of the most dangerous errors in morals. Under such influences, the national spirit became misdirected and its taste perverted. The Spaniards began to esteem nothing but bombast and inflation, both in conversation and in writing. Wise and patriotic, then, was the design of Cervantes, in overthrowing the whole fabric of chivalric literature. He did not make the attempt by the aid of arguments, grave dissertations, sermons, and legislative prohibitions—his weapons were satire and ridicule, and with these he was eminently and entirely successful. The romances of chivalry ended with "Don Quixote."

That we may not give our readers time to ask, what bearing have these remarks on the question of Cervantes being received into the ranks of the medical profession, and of his "Don Quixote" finding a place in the medical history of Spain? we shall go to the pith of our subject without further delay. Everybody knows that Cervantes has written the history of a madman, but it was reserved for the inquiring spirit of a countryman, Dr. Antonio Hernandez Morejon, to point out the kind of madness with which Don Quixote was affected, and to show how accurately its distinctive features have been delineated. This writer, at the conclusion of his Bibliographical History of Spanish Medicine,* claims for Cervantes a more accurate power of description of insanity than was displayed even by Aretæus.† In following the line of argument pursued by M. Morejon in support of his opinion, we shall avail ourselves of a French translation by Dr. J. M. Guardia, who has made some additions in the way of introduction and commentary.

The Spanish physician refers to the well-known fact, that every hospital and asylum holds within its

* *Historia Bibliografica de la Medicina Espanola.*

† Bellezas de Medicina Practica descubiertas en la Obra de Cervantes, pp. 166–180.

walls a maniac who believes himself to be some great personage—king, pope, general, a rich and powerful man, or a saint, and even the Deity himself. But, he adds, we nowhere find, in the annals of insanity, a madman so extraordinary, so benevolent, so amorous, so intent on advancing the public good as the wandering knight, who was desirous of driving out of the world all rogues, scoundrels, and wicked persons, and of redressing all the wrongs and iniquities which they had committed, as well as of pouring a healing balm on the pains, sufferings, and labors of the unfortunate; in a word, the hero who was to disenchant the peerless Dulcinea del Toboso. Let us now analyze the insanity of Don Quixote under its various aspects.

"Cervantes proposed to himself to describe a peculiar species of insanity. He begins by studying the manner of life and habits of the person, and the nature and character of the affection which he is about to draw, giving in their entireness all the predisposing and occasional causes which would be most likely to contribute to its development. He indicates its seat, and passes in review its periods, without losing sight of its changes and termination; he reasons on the prognosis, adopts the most suitable means of treatment, in such strict conformity with the rules of art that he may serve as an example to all philosophical physicians."

Bringing under notice the predispositions and causes of insanity, we meet with —1. "A bilious, melancholic temperament—Don Quixote was of lofty stature, with a complexion indicating robustness; his face was thin, body lean and hairy. 2. Adult or mature age—Don Quixote was bordering on fifty years. 3. An acute and cultivated intellect—Don Quixote had intelligence, an excellent memory, and large attainments; he had all the knowledge claimed for a knight-errant; theology, jurisprudence, medicine, botany, astronomy, mathematics, history, etc.—a most learned madman, beyond dispute, when his maniacal turn came. 4. The pride of ancestry and nobility—Don Quixote was a gentleman (*hidalgo*) of La Mancha, descended in a direct male line from Gutierra Quijada, the conqueror of the sons of the Count St. Pol. 5. Violent exercises—Don Quixote was a great hunter of hares. 6. The transition from an active to an idle life—Don Quixote neglected almost entirely the exercise of hunting, and even the superintendence of his estate. 7. Highly seasoned and nutritive food and that difficult of digestion—Don Quixote ate most commonly at his supper meat-hash, lentils on Friday, flesh meat on Saturdays, and young pigeons on Sundays. 8. Summer and autumn—Don Quixote suffered from his worst paroxysms of insanity on the 28th of July, the 17th of August, and the 3d of October. 9. Amorous passions—Don Quixote was deeply in love. 10. Excessive reading—Don Quixote sold many acres of his arable land in order to buy works on chivalry and erotic poems. 11. Prolonged wakefulness—Don Quixote 'read without ceasing day and night, to such a degree that by reading and sleeplessness, other causes contributing, his brain became dried up, so that he lost his judgment.' We find designated in this last sentence, with all the precision and clearness that could have been displayed by Hippocrates and Boerhaave, the diseased organ, the seat and immediate cause of the malady."

*Symptomatology.*—When once Don Quixote had become deranged, he imagined that all that he had read in books of chivalry and erotic poems were realities. His imagination ran on quarrels, battles, challenges, wounds, declarations and offers of love, and other extravagancies. Under the firm belief that all the absurdities met with in his reading were historical realities, he conceived the idea of becoming a knight-errant and to roam the earth in search of adventures. Dr. Morejon calls the madness of Don Quixote, monomania—his translator, Dr. Guardia, says no—it was many-sided mania. To our mind it seems that the dominant and persistent conviction of the Don, throughout, that he was a true knight-errant, who had vowed to redress the wrongs and succor the oppressed, was certainly that of a monomaniac; of one who suffered at the same time from hallucinations and illusions, in taking inns for castles and windmills for supernatural agencies, and ordinary persons on the road for oppressors, magicians, and other objects of a diseased imagination. The mind of Don Quixote was not the victim of complete mania. Apart from his dominant delusions of his vocation as knight-errant, when he was indeed haughty, arrogant, combative, daring, and rash, he showed himself on other occasions to be not only kind, courteous, and polite in his deportment, but, also, abounding in philosophical reflections and discriminating judgment. On all subjects he reasons admirably; he utters eloquent dissertations, and is fitter, as Sancho says, "rather to be a preacher than a knight-errant." The fine traits of mind and character, and even the extravagancies and insane delusions of Don Quixote, are brought out in stronger relief by contrast with the sensuality, gluttony, idleness, cowardice, and cunning, and at the same time a dash of shrewdness and homely wit of Sancho.

"*The Duration and Periods of the Disease.*—All diseases, without exception, whether they be long or short, have their periods or stages. Cervantes was careful not to make his patient an exception to this law laid down by Galen; the beginning, the increment, the height, and the decline of the insanity are indicated formally in his work, by so many escapades of Don Quixote.

"It began in the summer and declared itself in this fashion; the knight spoke to himself in his room on things relative to chivalry, and analogous to the occasional causes of his disease. Sword in hand, he made lunges at the walls, as if attempting to overcome giants, felons, and outlaws, and to obtain a triumph over them, and to redress wrongs, and procure satisfaction for misdeeds and injuries.

"Next he set about preparing all kinds of arms, and conceived the project of roaming over the land, in the exercise of the profession of knight-errant—a project which he carried out by his escapade of the twenty-eighth of July, one of the hottest days of the season, and on the night of which he exhibited the first paroxysms of the furor and recklessness of his insanity, followed soon afterward by the meeting with the half naked boy tied to the trunk of a tree, and with the merchants of Toledo.

"The increment of the disease is manifested from the date of the second sortie of the debonair cavalier from home, up to the time of his return. During this interval occurred the adventure of the windmills, the combat between the hero of La Mancha and the Biscayan, the melée with the bloody-minded Yanguesses, the adventures in which an inn is mistaken for a castle, and those of the funereal convoy, the fulling mill. Mambrino's helmet, the deliverance of the galley slaves, the penance in the wilds of the Sierra Morena, the assault on the bags of red wine, and the dealings with the archers of the Holy Brotherhood, and the white penitents. In the recital of this period of the increment of the disease, Cervantes secures beyond dispute the admiration of every philosophical physician. In this part of his work, he has, to my mind, says Dr. Morejon, drawn that species or variety rather of mania, of which Aretæus, at the end of a chapter devoted to the subject, has said: 'There exists another kind of delirium in which the afflicted lacerate their bodies, in the pious belief that the gods so will it and look with favor on such acts.' The picture which the Spanish author has drawn of the mania of Don Quixote, Knight of the Sorrowful Countenance, surpasses the original of the physician of Cappadocia."

In this picture Cervantes has united all the traits which indicate the greatest intensity of this disease, viz., incredible endurance of continued vigils, prolonged and alarming abstinence from food, insensibility to the action of cold, profound sighs, tears, fervent prayers, decided desire to tear and divest himself of his clothing, retaining nothing but his shirt; to make whirls and summersets; an enormous display of muscular and nervous tension of the nerves and muscles, and mortifying the flesh in honor of the goddess of his love, the peerless Dulcinea.

"In his retreat in the Sierra Morena, we are struck with a circumstance worthy of special notice; it is the meeting with Cardenio. For the most part, the insane prefer isolation: shunning each other's company, and despising and ridiculing one another: the only sympathy manifested among them is when their delirium is of the same nature. It is precisely this fact which Cervantes has noted with a master hand, in the episode of this gallant young fellow, who had become insane under the belief that Fernando had carried off his idol, Lucinda. On this occasion we have an example of those lucid intervals which are constantly exhibited by the insane. Cardenio's recital of his misfortunes to the curate, in one of these intervals, deserves to be read as illustrative of this truth. Another trait which merits the notice of medical men is the practice of maniacs to change their names. During the period in question, Don Quixote took the title of the Knight of the Sorrowful Countenance, and, at a later period, that of Knight of the Lions.

"The shades of alternations of the moral character in monomania are, pride, disdain, an exaggerated feeling of personal importance, and confidence in his own prowess. Don Quixote was continually boasting of the strength of his untiring arm; and on one occasion he went so far as to tell his squire that heaven had not created nor hell ever seen any one capable of inspiring him with fear.

"The last sortie of our hero, ending with his defeat by the Knight of the White Moon, after which he returned home for the third time, includes the periods of the height and the decline of his insanity. The marked symptoms were exhibited in the adventures with the wain of the Parliament of Death; the Knight of the Mirrors; the lions; in the cavern of Montesinos; the famous adventure of the enchanted boat; that of the afflicted duenna; the unequal combat with Tosilos; the battle with the bulls; the adventure of the beautiful Moor; that of the swine; of the enchanted head; and finally that of the Knight of the White Moon, at which begins the transition of one disease to another, a transition called by the Greeks *metaptosis*, and which is one of the most curious and difficult subjects in practical medicine.

"*Transformation of Insanity.*—Diseases sometimes extend from one organ to another, without any abatement of the primitive lesion; or they vary in their manifestation, although the organ primarily affected still exhibits the symptoms of the disease. Cervantes gives us an instance of this transformation of the malady. In Don Quixote's case an acute fever supervenes, and immediately all the physical and general features of the first affection are altered. These changes give rise to curious inquiry, in three ways: first, in relation to practical medicine; secondly, as applied to medical jurisprudence, for without this change Don Quixote could not have made his will, or if he had done so, it would have been null and void; and, finally, in relation to the effect of the change in the disease on its prognosis and termination.

"The sudden conversion of the insanity into a fixed depression, and a profound melancholy; the complication of a violent fever, and the sudden transition from madness to reason, were so many circumstances which ought to inspire fears for the life of the sick man; and it was precisely this series of phenomena which served as presages to the death of the celebrated knight."

Dr. Morejon claims for the Spaniards, his countrymen, the honor of the first recourse to the humane and mild treatment of the insane, the initiation of which in France, and, generally, as it was thought in Europe at large, constitutes the chief title of Pinel to fame. The rational method adopted in the insane hospital at Saragossa, and praised by Pinel, was probably borrowed from that of Valencia. It was exhibited in practice, two centuries before the time of the French author, by Cervantes, in a formal manner, with equal genius and skill. One cannot but admire the medico-moral strategy which he brought to bear in order to calm the fury and ravings of his knight-errant; the means were not less original than that by which he banished from Spain the bad taste that induced everybody to read books of chivalry. Cervantes brought with him to his task a large and varied experience of the world, in which he had acted many parts, a keen insight into human nature, with whose infirmities and vagaries he had formed an intimate acquaintance. Cervantes had made himself entirely master of the character of Don Quixote, as of a living and acting man, one whom he came to look on as his son, and, as such, one who had become the object of his tenderest care in devising the means for the relief of his distressing malady.

"Six persons figure in different characters in this history, as taking a part in the treatment, to meet the two extremes of the epigraph of Boerhaave: the curate, a well-informed man, master Nicholas, and Sanson Carrasco favor the absurdities of the knight; the canon of Toledo, the governess, and the niece oppose them with energy."

It was necessary to begin the treatment by a removal of its efficient cause, and hence an examination and burning of all the books on chivalry and love, which had been shut up in a separate apartment closed by a wall, and then the persuading the patient that the destruction was the work of enchantment. This was the most sensible course which could be pursued under the circumstances of the case. The learned magician Mugnaton, mounted on a serpent, comes in a cloud; he escapes by the roof, and leaves the house filled with smoke. The cure was, however, retarded, partly on account of the plan of the romance in the mind of the author, who could not allow the story to end thus languidly, and in part, as Dr. Morejon points out, owing to a slight inadvertence of the niece, who confounded the name of Freston or Friston with that of Mugnaton,—an instance intended to show the necessity of great sagacity and prudence in such cases, as the least negligence will upset the best-devised plans.

By the devices of the curate and the village barber, who enlisted the fair Dorothea on their side, the crazy knight is persuaded to leave his solitude in the Sierra, and is brought to a tavern, where he falls into a profound sleep blended with a kind of somnambulism, which was a prelude to a remission of the disease. In this state he is carried, without much resistance on his part, to his home. The resolve of the curate and barber to let an entire month pass without seeing the sick man, for fear of awakening the memory of past occurrences, now that he began to exhibit proofs of returning reason, was excellently conceived: it would have been still better if all the members of his family had stayed away. The dietetic regimen prescribed and followed in the cure was most appropriate. When the disease was on the point of reappearing, the threats of his housekeeper, that she would complain loudly both to God and the king, if he did not remain quietly at home; those of his niece, when she pointed out to him that all his narratives of knight-errants were fabulous and false, and were only fit to be burned, and at the very least merited the censure of the Inquisition,—these threats and complaints were among the best adapted, and in Spain the most powerful, to arrest the insane outbreak. As such they were turned to account by the canon of Toledo.

The third device of a curative nature was the agreement between the curate and the barber, in conjunction with the bachelor Sanson Carrasco, who disguises himself as the Knight of the

Mirrors and engaged in single combat with Don Quixote. The same plan was followed out to the approaching termination of the disease, during the time when he had become a shepherd. This moral treatment of the insanity of Don Quixote was attended with an abatement of the disease, which is as exactly and truly described by Cervantes, as if he had borrowed the pen of Aretæus himself.

Not only did Cervantes precede Pinel in the moral treatment of insanity, but also, adds Dr. Morejon, Broussais himself in the doctrine which gained the latter so many proselytes in Europe; for the Spanish author, in laying down the axiom, through the saying of the crazy man of Seville, that "the stomach is the laboratory in which health is fabricated," shows thereby the connection between this organ and alterations in the judgment. The reveries of Hahnemann were also anticipated by Cervantes in a practical and common sense manner: first, in the conception of his great work "Don Quixote," to cure epidemic insanity, arising from knight-errant literature, by the fictitious history of an insane man; and, secondly, to relieve his patient by appeals to his senses and imagination, similar to those by the long reading and study of which he had first become distraught.

Determined to carry out the clinical study of Don Quixote to the end, Dr. Morejon sees only one omission in the great work of Cervantes to interfere with its completeness: this was the failure to make a *post mortem* examination of the famed knight of La Mancha. But he finds plausible reasons for this omission, some of which are purely speculative, if not imaginary. The true one was, doubtless, the impossibility of an examination being made, owing to the prejudices of the family and near relations of the deceased, especially in a village,—prejudices, we may add, common enough both in village, town, and city, even in this our present age of boasted enlightenment.

In conclusion, we cannot withhold from Dr. Morejon the credit of having added a new leaf to the laurel crown long awarded to the author of the inimitable "Don Quixote." He has accumulated proof upon proof of the medical instinct, as we might term it, certainly of the powers of medical observation, of Cervantes. We must not suppose, however, that this great genius was indebted to his imagination alone for the admirable picture of insanity which he has drawn. Scholar, soldier,—fighting and losing a hand at the battle of Lepanto,—traveler, long time a prisoner among the Moors in Algiers, and whose life was marked by many adventures abroad and of vicissitudes at home, he could not have passed by with indifference either the individual case of insanity met in chance rencontre, or the collection of many persons in insane asylums, in the different places which he visited, and especially those of his own country.

"Don Quixote" was not the only portrait of a deranged man drawn by Cervantes. In one of his many moral novels, as he called them, he introduces to his readers a poor devil of a licentiate, Vidriera by name, who had spent his youth in study amid books, at the schools, long resisting the seductions of love, until he was poisoned by a philter, which threw him into a lingering malady, accompanied by mania. The monomania in this case consisted in the belief of the licentiate that his body was made of glass; and hence an account of the most minute and comic precautions for preserving this frail structure from the rude contact of external objects.

The reading of "Don Quixote" in a medical sense, while it increases our admiration for the genius and descriptive powers of the author, can hardly fail, at the same time, to temper the exuberance of spirits which would otherwise be induced by a perusal of the narrative of the ludicrous situations in which the knight and his squire are thrown, and of the comic incidents of which these personages are the occasion. Even before the enlargement of the field of our mental vision by this new light thrown on the subject, most readers, of ordinary reflection, must find themselves in a state of feeling of a secret interest in the successful issue of the adventures of the madman, while laughing at his extravagancies; nor can they separate, in their minds, the amusement derived from the absurdities displayed during the paroxysms of his insanity from the respect and deference which they feel for the good knight in his lucid intervals. The raving mania is, on these occasions, replaced by pleasing evidences of good education and gentlemanly refinement, increased powers of memory, witty sallies, and *bon mots*. It is thus that the Don shows himself in the palace of the duke, and at the house of Don Antonio Moreno, at Barcelona. So, also, in his narratives and conversation, in his catches, and in the episodes which embellish the work, we meet with lessons and precepts for all classes of society.

